# Exploring the illness experiences amongst families living with 2019 coronavirus disease in Ghana: Three case reports

**DOI:** 10.4102/phcfm.v12i1.2682

**Published:** 2020-11-06

**Authors:** Nana K. Ayisi-Boateng, Dora Egblewogbe, Ruth Owusu-Antwi, Akye Essuman, Kathryn Spangenberg

**Affiliations:** 1 Department of Medicine, School of Medicine and Dentistry, Kwame Nkrumah University of Science and Technology, Kumasi, Ghana; 2 Department of Polyclinic/Family Medicine Sub BMC, Korle Bu Teaching Hospital, Accra, Ghana; 3 Department of Psychiatry, Komfo Anokye Teaching Hospital, Kumasi, Ghana; 4 Family Medicine Unit, Community Health Department, University of Ghana Medical School, Accra, Ghana; 5 Family Medicine Directorate, Komfo Anokye Teaching Hospital, Kumasi, Ghana

**Keywords:** biopsychosocial, COVID-19, family, Ghana, illness experience

## Abstract

The 2019 corona virus disease (COVID-19) has wreaked havoc on countries, communities and households. Its effect on individuals and their families, although enormous, has not been adequately explored. We thus present a report on the illness experiences of three families in Ghana who had at least one member diagnosed with COVID-19. We interviewed them and recorded their commonest fears, such as death, stigmatisation and collapse of family business. Respondents had a fair idea about symptoms of COVID-19, mode of transmission and safety precautions. Family separation and loss of income were some of the adverse effects expressed. Majority of them were hopeful that family members with COVID-19 would recover and be reunited. The biopsychosocial impact of COVID-19 is tremendous and family physicians and other primary care workers have an essential role to play in addressing this.

## Background

Ghana detected her first two cases of the 2019 coronavirus disease (COVID-19) on 12 March 2020,^1^ a day after the World Health Organisation (WHO) declared it a pandemic. Subsequently, the global spread has been phenomenal and, as of 16 August 2020, the number of infected individuals reported is 21 294 845 while the number of reported deaths is 761 779 (case fatality ratio 3.6%).^2^ In Ghana, there are 42 210 confirmed cases and 231 deaths (case fatality ratio 0.55%).^2^ The rapid spread of the infection and high mortality cause anxiety, worry and fear, defined as an unpleasant emotional state that is triggered by the perception of threatening stimuli.^3,4,5^

The COVID-19 pandemic has had unprecedented impact on global public health and on several other aspects of modern society.^6^ Governments’ restriction measures, lockdowns and isolation of infected persons place huge economic burden and stress on families.^7^ People living in under-resourced countries are most vulnerable. Current literature has focused on the biomedical impact of the pandemic with little attention to the psychosocial effects, especially on families living with COVID-19. We therefore present a report on the illness experiences of three families in Ghana that had one or more members diagnosed with the disease.

### Family 1

A 52-year old male trader with a travel history to Europe, presented to the University Hospital, Kwame Nkrumah University of Science and Technology (KNUST), Kumasi, Ghana on 07 April 2020 with fever (temperature 39 °C) but no respiratory symptoms.

A nasopharyngeal sample was taken and his result was positive for COVID-19. The homecare team visited him in appropriate personal protective equipment (PPE), obtained his consent to counsel the family, disclose his test results and test the rest of them. The index patient lived with his wife, three children, all of whom tested negative for COVID-19, and a 24-year old female domestic caretaker, who tested positive. She was asymptomatic and her temperature was 36.3 °C. The family lived in their own private accommodation where each person had access to private bathroom facilities. Based on the guidelines by the Ministry of Health, Ghana,^8^ they were eligible for home-based care.

### Family 2

A 24-year-old male health worker, reported to the University Hospital, KNUST, with a 2-day history of rhinorrhoea, sore throat and headache on 28 May 2020. He tested positive for COVID-19. A team visited him at home, where he lived with his parents and eight other family members. With his consent, the test report was disclosed to them and they were counselled. He acquiesced to be transferred to the regional isolation centre. Oropharyngeal samples were taken for all members of the household. Four of the family members tested positive for COVID-19 but were asymptomatic. All the regional isolation centres were full and the family opted to isolate at home for 14 days. The family did not have separate bathroom facilities so six family members who tested negative relocated to stay with other relatives. The family’s merchandise shop had to be closed during this period of isolation.

### Family 3

A 41-year-old building artisan, who suspected exposure to an individual confirmed with COVID-19, reported to the University Hospital, KNUST on 1 June 2020 for screening. He tested positive. His 14-year old daughter tested positive but his wife and two other daughters tested negative. All of them were asymptomatic. They lived in a densely populated house and shared kitchen and bathroom facilities with other tenants. He and his daughter were transferred to the regional isolation centre, whilst his wife and the other daughters stayed at home.

## Method

All families selected had at least one member test positive for COVID-19. Those selected were either asymptomatic or had mild symptoms and their clinical management was based on the Ministry of Health protocols.^8^ They were interviewed during the course of 14-day isolation. They were all followed up till discharge or repeat tests were negative for COVID-19. Family members who were < 18 years of age were not interviewed. Employing a validated tool,^9^ a family physician and a psychiatrist explored their illness experiences as follows:

1. What are your *fears* about COVID-19 in your family?
2. What are your *ideas* about the infection?
3. How has the infection in your family affected your regular activities or *function*?
4. What are some of your *expectations*?

## Discussion

This report highlights the unique contextual dynamics associated with families living with COVID-19 in Ghana. A family-centred, multidisciplinary team-based approach was employed to address their challenges. The team included family physicians, community-based disease control officers and a psychiatrist. During the period of managing the families, the number of confirmed COVID-19 cases in Ghana soared with attendant pressure on healthcare facilities and few available isolation centres. We had to adopt telemedicine (regular calling of patients on phone) and home-based care for asymptomatic or mild cases and tracing of contacts in the community. As observed in Family 2 and Family 3, there were feelings of loneliness, sadness and alienation as a consequence of family separation. This required counselling, psychotherapy and expedited repeat COVID-19 tests to facilitate discharge of confirmed cases and early reunion of families.

Whilst common symptoms of COVID-19 include fever, cough and other respiratory manifestations,^10^ majority of the confirmed cases in this report had no symptoms. This largely asymptomatic picture of COVID-19 resulted in denial, doubt about the diagnosis and regret for testing amongst some of the respondents (Table 1). Fear of infecting close family contacts, death, stigmatisation and loss of income were expressed and these have been reported elsewhere.^3,11,12^ Some fears, if not addressed, could lead to suicide and post-traumatic stress disorder.^11,13^ Interestingly, in Family 1, isolation at home for 14 days engendered family unity and love (Table 1). The affected families were hopeful and their expectations were generally positive.

**TABLE 1:** Summary of family responses on their fears, ideas, function and expectations of the COVID-19 pandemic.

Families and COVID-19 status	Fears	Ideas	Function	Expectations
**Family 1**				
Index patient – father (positive)	Death, stigmatisation, business collapse. Family members getting infected.	Physical contact promotes infection. No cure yet. Symptoms include fever and respiratory symptoms.	Impaired social interaction. Loss of income.	Discovery of cure. Prevent stigmatisation. Financial support.
Wife (Negative)	Getting infected.	Rapid spread if precautions are not taken.	Loss of income. Home isolation engendered family unity.	Cure for COVID-19.
Domestic caretaker (positive)	Death, stigmatisation.	Isolating the infected person can limit community spread.	Loss of friends.	Vaccine production. Establishment of COVID-19 treatment facilities.
**Family 2**				
Index patient – son (positive)	Collapse of parents’ business. Stigmatisation.	Doubts about test results. Regret testing.	Emotionally shattered. Loss of income.	Be discharged to reunite with family.
Father (positive)	Worsening symptoms.	Health workers at high risk of infection.	Loss of income.	Expedited retesting.
Mother (positive)	Possible ineffective precautionary measures.	Doubts about test results.	Depleted family resources.	Negative retest results.
Grandmother (negative)	Death of family members who tested positive.	Difficulty in understanding asymptomatic COVID-19.	Insomnia. Difficulty re-adjusting after relocating to another house.	Prompt recovery of affected family members.
**Family 3**				
Index patient – Husband (positive)	The 2-year old child getting infected.	Infection can be prevented.	Family separation.	Discovery of cure. Speedy recovery of family members.
Wife (negative)	Family members getting infected.	Safety precautions are effective.	Family separation.	Early recovery of family members.

## Conclusion and recommendation

Families living with COVID-19 experience psychosocial challenges in addition to physical and economic challenges. Family physicians should lead efforts to address these challenges by galvanising team work and the use of telemedicine during the pandemic to enable constant and coordinated virtual – but personal – follow-ups whilst at the treatment centres and at home. They should also employ relevant family medicine tools whilst caring for patients with COVID-19 and advocate for the protection of the most vulnerable families. Potentially, this will assuage their fears, minimise effects of family separation and diminish stigmatisation.
